# What do people with aphasia want to be able to say? A content analysis of words identified as personally relevant by people with aphasia

**DOI:** 10.1371/journal.pone.0174065

**Published:** 2017-03-27

**Authors:** Rebecca Palmer, Helen Hughes, Tim Chater

**Affiliations:** 1 School of Health and Related Research, University of Sheffield, Sheffield, United Kingdom; 2 Sheffield Teaching Hospitals NHS Foundation Trust, Sheffield, United Kingdom; Kobenhavns Universitet, DENMARK

## Abstract

**Background:**

Word finding is a common difficulty for people with aphasia. Targeting words that are relevant to the individual could maximise the usefulness and impact of word finding therapy.

**Aims:**

To provide insights into words that people with aphasia perceive to be personally relevant.

**Methods and procedures:**

100 people with aphasia were each asked to identify 100 words that would be particularly important for them to be able to say. Two speech and language therapist researchers conducted a quantitative content analysis of the words selected. The words were coded into a framework of topics and subtopics. The frequency with which different words and topics were selected was then calculated.

**Outcomes and results:**

100 participants representing 20 areas of the United Kingdom ranged in age from 23 to 85 years. Word finding difficulties ranged from mild to severe. The sample of 9999 words selected for practice included 3095 different words in 27 topics. The majority of words selected (79.4%) were from the topics ‘food and drink’ (30.6%), ‘nature and gardening’ (10.3%), ‘entertainment’ (9.4%), ‘places’ (7.3%), ‘people’ (6.7%), ‘house’ (6.5%), ‘clothes’ (5.2%) and ‘travel’ (3.5%). The 100 words types chosen with the greatest frequency were identified. These account for 27 percent of the 9999 words chosen by the participants.

**Discussion:**

Personally relevant vocabulary is unique to each individual and is likely to contain specific or specialist words for which material needs to be individually prepared. However there is some commonality in the words chosen by people with aphasia. This could inform pre-prepared materials for use in word finding therapy from which personally relevant words could be selected for practice.

## Introduction

Approximately a third of people have aphasia after a stroke. Aphasia affects different domains of language including understanding spoken language, reading, writing and speaking to varying degrees. Word retrieval difficulties are common to the majority of people with aphasia and contribute to making communication difficult and frustrating. Consequently speech and language therapy often focusses on word finding with the aim of increasing the ability to retrieve words in everyday communication, to improve verbal interactions and reduce frustration [1].

Therapy approaches for the retrieval of single words are often subdivided into phonological therapies and semantic therapies. Phonological therapies may use word repetition, phonemic cueing or orthographic (written whole word or letter) cueing. Semantic therapy techniques usually focus on word to picture matching or describing the properties of a target word, for example what it does or what it is used for. Cues and descriptions can be provided by the therapist to prompt word retrieval, or the individual can be encouraged to self-cue [2]. Cues are often presented in a hierarchy of difficulty until the participant retrieves the word successfully, starting with a phonemic cue and building up to presenting the whole word to be repeated [3]. Intensive language action therapy (previously known as Constraint Induced Aphasia Therapy) targets the use of words to make requests in increasingly complex phrases and sentences. This moves beyond word retrieval in isolation to word retrieval in functional language tasks [4,5]. Word finding interventions have been delivered during face to face treatment sessions with a speech and language therapist using table top/paper based therapy materials and within specialist computer therapy programs and apps [6–8]. Common to all therapy techniques and modes of delivery is the use of pictures and written words/letters as stimuli for word retrieval.

Therapy has been shown to be effective for words used within therapy sessions (treated items). For example, Best et al 2013 showed 15/16 participants improved retrieval of treated items in a cueing hierarchy therapy delivered face to face [3]. Palmer et al (2012) showed improvement of treated items in 10 out of 15 participants following a self-managed computer word finding therapy in a pilot study using the StepbyStep program [8,9]. Speech and language therapists deliver therapy with the intention of having an impact on the individual’s everyday communication outside of therapy. This assumes generalisation of the word finding improvements seen in therapy. Generalisation encompasses two key ideas. The first is that words learned in therapy are used in everyday functional communication situations and that following therapy the experience of communicating is improved. Best et al (2008) found an improvement in both word finding and patient rated activity levels using the Communication Disability Profile [10] following impairment based word finding therapy [1]. During interviews with participants and carers of participants who had used StepbyStep computer word finding therapy, participants gave examples of using words learned in therapy in everyday functional communication contexts. In addition participants and carers described an increased confidence to participate in social situations where communication is required [11]. The second meaning of generalisation is that the word finding improvement shown for words treated in therapy is also shown for untreated items. In a review of word finding studies Nickel et al 2002 showed that approximately one in four people with aphasia generalised improved word finding of treated items in therapy to untreated items [2]. More recently, Best et al 2013 also showed that whilst 15/16 participants improved naming of treated items, 4/16 (1 in 4) improved retrieval of untreated items by more than four percent [3]. Common to all four participants who showed generalisation to untreated words was that their word finding difficulties were phonological in nature. Those with semantic difficulties or mixed semantic and phonological difficulties did not generalise to untreated items [3].

Best et al (2013) highlight the importance of using functionally relevant words in therapy for people with aphasia for whom therapy does not always generalise well to untreated items [3]. Neuroplasticity theories describe the process by which re-learning of skills occur following brain damage. They provide further support for treatment of words that are of particular interest or use to an individual by identifying salience as an important factor in treatment success [12]. In other words, meaningful learning environments or materials may enhance re-learning. Participants and carers who took part in the pilot CACTUS study which focussed on practising personally relevant words, highlighted that making sure that the practice material was of interest to them was motivating [11].

To maximise the impact of word finding therapy on the lives of people with aphasia, it is important to, ensure that words selected for use in therapy are of personal relevance to the individual being treated. Preparing a bespoke set of picture material for each individual can be time consuming and therapists often rely on pre-prepared colour flashcards or libraries of images within computer programs or apps. In order for these pre-prepared resources to be of maximum use to therapists, they need to contain pictures of words that people with aphasia commonly identify as being of personal relevance to learn. But what do people with aphasia see as relevant?

In the Big CACTUS trial of computerised word finding therapy all participants with aphasia were asked to select 100 words that they thought would be particularly useful for them to be able to say. They were then randomised to computer therapy with the StepbyStep program for 6 months, an attention control group (completion of puzzle books), or usual care [13]. A picture was found to represent each word chosen in order to provide stimuli for baseline and outcome word finding tests and, if randomised to the computer therapy group, to provide stimuli for the treatment of the 100 words for the six month intervention period. This study analysed the words chosen by 100 participants in the Big CACTUS trial to provide insights into what people with aphasia perceive to be personally/functionally relevant.

The study answers two questions: 1) What are the topics that people with aphasia are interested in talking about? 2) What are the most common words and topics selected for practice by people with aphasia?

## Methods and materials

### Design

A quantitative content analysis was used to analyse lists of personally relevant words generated by 100 people with aphasia. Analysis followed the six stages of content analysis proposed by List (2007) [14]: 1) selecting content for analysis, 2) identifying the units of content, 3) preparing content for coding, 4) coding content, 5) counting and weighting, 6) drawing conclusions.

### Participants

People with aphasia post stroke were recruited to the Big CACTUS trial from twenty National Health Service (NHS) trusts across the United Kingdom (UK) [13]. Participants were included if they were aged over 18 years; had a diagnosis of stroke at least four months prior to randomisation; had a diagnosis of aphasia subsequent to stroke; were able to retrieve 10–90 percent of words on the Comprehensive Aphasia Test Naming Objects subtest [15]; were able to perform a simple matching task in the StepbyStep computer program with at least 50 percent accuracy; and were able to repeat at least 50 percent of words in a StepbyStep repetition task. Participants were excluded if they had another pre-morbid speech and language disorder caused by a neurological deficit other than stroke; required treatment for a language other than English; were currently using the StepbyStep computer program or other computer speech therapy aimed at word retrieval/naming.

Recruitment was carried out by speech and language therapists trained by the Big CACTUS research team. The Consent Support Tool [16] was used to ensure informed consent was taken where possible. When a potential participant did not demonstrate capacity to provide consent, their inclusion was enabled by a signed consultee declaration of belief that their friend or relative would like to participate. See Big CACTUS protocol [13] for further details. The study protocol was approved by Leeds West National Health Service research ethics committee [reference 13/YH/0377]. Additional approval was granted for Scotland by the Scotland A research ethics committee [reference 14/SS/0023].

### Procedure for selecting personally relevant words

After participants had consented to the Big CACTUS study, the speech and language therapists asked them to choose 100 words that they would find useful to be able to say. They were given time to think about this with their families. In addition, 18 picture cards were designed to show participants possible topic areas to prompt them to think about what was important to them. The cards were informed by topics chosen in the pilot CACTUS study [8] and the content of the StepbyStep program library [9]. Once a set of 100 words had been chosen they were grouped according to words related to the same topic of interest and entered onto a central electronic project database. Pictures were identified to represent all of the words chosen for each participant to be used in personalised word finding tests for all participants and for therapy for those randomised to the computer therapy group.

### Content analysis procedure

The purpose of the analysis was to identify topics of interest to people with aphasia and to identify the most common words and topics selected for therapy by people with aphasia. The analysis was conducted by two speech and language therapy researchers familiar with the StepbyStep word finding therapy and the approach taken to word selection.

Selection of content for analysis: Word lists from the first 100 participants randomised to the Big CACTUS trial were selected for analysis. This number of participants ensured word lists from participants with a range of ages and genders from all 20 areas of the UK participating in Big CACTUS were represented. All words in word lists from the first 100 participants were analysed.

Units of content: Single words or terms made up of two or three words (e.g. ‘cup of tea’) were the units of content in this analysis.

Preparing the content for coding: The words from all 100 participants were listed in one document, S1 File. Terms that contained more than one word were then joined and hyphens and other special characters (such as apostrophes, question marks) were removed to ensure the term was analysed as one unit of content (for example ‘tool-box’ was changed to ‘toolbox’, and ‘cup of tea’ to ‘cupoftea’). Each of the words chosen by each participant was considered as a word token. The SLT researchers classified all the word tokens into word types. All tokens of one word type were labelled with the most frequent token used. For example ‘banana, banana, banana, bananas, bananas’, chosen by five different participants would represent five tokens of the word type ‘banana’. As the most frequent token is ‘banana’ all tokens of this word type were labelled as ‘banana’. Another example is that word tokens ‘tea’ (the drink) and ‘cup of tea’ were classed as one word type ‘cupoftea’. The content was prepared by one research speech and language therapist and checked by the other to identify any omissions such as hyphens left in or tokens of words unaccounted for. The transcript was then imported into NVivo 10 software which was used to present a list of word types and the number of tokens per word type in alphabetical order.

Coding the content: The word types were coded into topics and subtopics forming a hierarchical coding framework in NVivo 10. The two researchers conducted this task until all of the words had been coded. The topics were defined by the researchers who decided what semantic category each word came from (for example ‘dog’ comes from the semantic category ‘animal’). One hundred and fifty three word types (five percent of the sample) were coded by both researchers independently. There was 76 percent agreement between the researchers on this sample. Once the data was all coded the researchers reviewed the coding together. To increase reliability, where the topic was ambiguous, the researchers referred back to the source data to provide context. For example the word ‘lamb’ could be coded as ‘food’ or as ‘animal’. If the source data showed ‘lamb’ was grouped with words such as ‘meat’, ‘chicken’, ‘beef’, it would be coded as food. Where the ambiguity was unresolved it was coded as ‘unable to identify topic’. Once the coding was agreed, the researchers identified where topics could be grouped to form overarching topics and selected labels for the overarching topics which described the content. A third researcher, familiar with the word selection method and content analysis method, was consulted where the two researchers did not reach agreement on a topic grouping or name.

Counting and weighting: Topic and subtopic labels were added to the original dataset of words chosen by individual participants (in other words each token of a word chosen by an individual participant was labelled with its topic and subtopic). See S1 File. This dataset was then the source for the quantitative analysis of the content which consisted of counts of the number of topics and subtopics and the number of word types in each topic and subtopic for the whole group of 100 participants.

The dataset was then used to identify the proportions of word tokens chosen from each topic by key subgroups and by the group as a whole. Subgroups explored included gender and age. As the total number of participants was 100, they were divided into only two age sub groups based on the frequently used clinical distinction of under 65 years of age or 65 years and over [17]. The percentage of words chosen from each topic was identified for the whole group and for each of the four subgroups (for example, the percentage of words chosen by men, which were from the topic ‘travel’). This enabled similarities and differences to be identified between subgroups. As one percent of the total number of words in the dataset is 100 words and each participant chose 100 words, it is possible that where one percent or fewer words were selected from a topic that these could all have been selected by the same participant. Comparisons were therefore only made for topics comprising more than one percent of the words. The topics were then ranked in order of frequency with which words from them were chosen, from highest to lowest for all subgroups. Box and whisker plots were created for the highest ranking topics to indicate the distribution of words chosen from these topics across participants.

The identification of frequently selected topics gives an indication of what people with aphasia may like to focus on in word finding therapy. However, it does not indicate which words from these topics are likely to be of interest to many patients receiving word finding therapy. To address this, the frequency with which each word type was selected was calculated and presented from highest to lowest. As 100 words were used in a set of therapy words in the treatment study from which these data arose, the 100 words chosen with the greatest frequency are presented in the paper for the group as a whole. Key differences between subgroups are also identified. Where there was more than one word selected with the same frequency in one hundredth place, all of these words were presented.

## Results

### Participants

Of the 100 participants, 63 were male and 37 were female. Word finding severity was based on the Naming Objects score of the Comprehensive Aphasia Test [15]. Scores of 5/48 to 17/48 were classified as severe, 18/48 to 30/48 as moderate and 31/48 to 43/38 as mild. In this sample 49 of the participants had mild word finding difficulties, 27 had moderate word finding difficulties and 25 had severe word finding difficulties. Participants were aged between 23 and 85 years with a median of 64 years. Fifty two participants were aged less than 65 years and 48 were aged 65 years or above. Table 1 shows the number of participants from each of the 20 areas of the UK involved in the study. The numbers vary depending on the length of time the area had been involved in the Big CACTUS study and the number of participants that had been recruited. S2 File shows demographics for each individual participant.

**Table 1:** Number of participants whose word lists were included in the analysis from each area.

Area	Number of participants
Ayr	1
Belfast	5
Cambridgeshire	3
Cwm Taf	3
Derbyshire	6
Dorset	3
Glasgow	12
Hull	8
Newcastle	6
Norfolk	4
North Lincolnshire	5
Northampton	4
Northern	5
Nottinghamshire	3
Plymouth	3
Sheffield	10
Somerset	2
South Bedford	12
Sunderland	1
Swansea	4

### Topics and subtopics of words

Three thousand and ninety five (3095) different word types were represented in the sample of words from the first 100 participants. (There were 9,999 words altogether as one participant only had ninety-nine words.) There was insufficient contextual information to categorise 104 of the words (Three percent of the sample). The remaining 97 percent of word types fell into 27 different topics shown in Table 2. Eighteen of the topics were further divided into subtopics shown in Table 2. A second level of subtopic was identified for 12 of the 120 subtopics. Eating and drinking out comprised generic names of places to eat out such as ‘restaurant’ or ‘pub’, specific names of places to eat out, and menus. Generic places included ‘city centre and town’ (for example ‘bank’); ‘coastal places’ including ‘beach’ and ‘seaside’; ‘exercises and fitness places’ (such as ‘gym’); ‘health and medical places’ (for example ‘hospital’); ‘public buildings and spaces’ (such as ‘church’) and ‘services’ (for example ‘post office’). Seven of the entertainment subtopics were further subdivided. Arts and crafts included both arts and crafts. Films comprised ‘genres’, ‘mediums’ and ‘modes’ (for example ‘DVD’), ‘production companies’ and ‘specific films’. Non sporting hobby words related to ‘hobbies’, ‘equipment’ used for these hobbies and ‘terminology’ used. Reading was subcategorised into ‘specific reading material’, ‘reference books’ and the ‘medium’ used such as ‘book’ or ‘kindle’. Sport words included those referring to ‘specific sports’, ‘sport equipment’, ‘league or competitions’, ‘players and referees’, ‘sports teams’ and ‘terminology’. Music words included ‘sound equipment’, ‘instruments’, ‘medium ‘of listening to music, ‘genre’, ‘shows’ and ‘songs’. TV words consisted of the category of programs (for example ‘documentary’), ‘channels’, ‘medium’ of watching TV (for example ‘iPad’) and ‘specific names of programs’. In nature and gardening animal words related to ‘types of animals’ and ‘looking after animals’ (for example ‘dog food’). Travel words relating to trips and holidays were subdivided into ‘accommodation’ (for example ‘hotel’), ‘relaxation’ (such as ‘sunbathing’) and the ‘type of trip or holiday’ (for example ‘cruise’). Vehicles included ‘makes’ (for example ‘Ford Focus’), ‘types’ (such as ‘car’) and ‘vehicle parts’(for example ‘engine’). Finally science words were categorised into ‘astrophysics’ (for example ‘telescope’), ‘elements’ (such as ‘hydrogen’), ‘materials’ (such as ‘leather’) and ‘general science words’ (for example ‘evolution’).

**Table 2:** Topics and subtopics of words selected for practice by people with aphasia.

Topic (Number of word types)	Subtopics (Number of word types)
People (488)	People’s names (283); Musicians (92); Sports personalities (41); Actors and TV celebrities (23); Relations (15); Fictional characters (13); Royalty (7); Authors (5); Religious figures (5); Comedians (2); Historical figures (2)
Food and drink (447)	Food (251); Drink (72); Equipment and utensils (57); ^a^Eating & Drinking out (22); Ingredients (15); Sauces and condiments (14); Types of cuisine (8); Meals of the day (4); Method of cooking (4)
Places (381)	Areas in British Isles (156); Places abroad (68); Specific public buildings and spaces in British Isles (56); Generic places (45); ^a^British landmarks (23); Specific roads and addresses in British Isles (14); Landmarks abroad (10); Sports venues (9)
Entertainment (377)	^a^TV (113); ^a^Sport (90);^a^Films (46); ^a^Music (38); ^a^Art & Crafts (32); Games, toys and puzzles (19) ^a^Non-sporting hobbies (18); ^a^Reading (10); Fitness equipment (6); Dancing styles (5)
Nature and gardening (279)	^a^Animals (146); Trees, plants and flowers (56); Tools and equipment (27); Looking after animals (16); Types of habitat (14); Garden surfaces (5); Boundary markers (3); Outbuildings (3); Outdoor seating (3); Soil and compost (3); Wood (2); Garden activities (1)
House (135)	Housework (28); DIY and tools (23); Soft furnishings (22); Furniture (18); Fixtures and fittings (14); Heating and lighting (12); Rooms (11); Types of accommodation (4); Outside the house (3)
Travel (110)	^a^Vehicles (66); ^a^Trips and holidays (15); Public transport, stops or stations (5); Travel documents and baggage (5); Commuting (4); Navigation (4); Roads (3); Fuel and filling up (3); Boating terminology (2); Disability parking (2); Flags (1)
Actions (98)	
Work and education (82)	Professions (62); Work related words (7); Education (5); Job specific equipment (3); Places of work (3); Companies (2)
Health (69)	Body parts (29); Medication (15); Disability and illness (12); Aids (6); Procedures (4); Therapy (2); Tests (1)
Shopping (69)	Specific shop names (21); Types of shop (16); Shopping centres and markets (14); Supermarket chains (8); Items associated with shopping (4); Online shopping (3); General places to buy things (2); Brands (1)
Clothes (67)	
Money and numbers (60)	Numbers (33); Cash amounts (12); Forms of payment (6); Financial products and savings (6); Places to withdraw cash (2); Currency (1)
Maths and science (58)	^a^Science (50); Maths (8)
Time (45)	Times (19); Months (12); Days (8); Seasons (3); Calendars and diaries (3)
Personal items (41)	Jewellery and cosmetics (28); Items carried around with you (10); Smoking related items (3)
Personal care (34)	
Descriptive terms (33)	General descriptive terms (19); Colours (14)
Organisations and groups (22)	Groups and clubs (8); Support groups (6); Regional and international organisations (5); Group admin processes (3)
Feelings and senses (19)	
Weather (17)	
Technology and equipment (16)	Hardware (8); Accessories (6); Make of equipment (1); Software (1)
Non content words (16)	
Communication mediums and modes (12)	
Stationery (6)	
Events (5)	Beer festivals (2); Goods related to celebrations (2); Celebrations (1)
Religion (5)	

^a^Subtopics that were further subdivided

### Topics chosen by subgroups

The numbers of word tokens selected from each topic are presented for the whole group and for each gender and age subgroup in Tables 3 and 4. The numbers of words chosen per topic are also shown as a percentage of all the words chosen for each subgroup. As there are different numbers of participants in each subgroup, the percentages of words chosen from each topic have been used to compare the subgroups.

**Table 3:** The number of words chosen from each topic by gender.

	Number of words chosen by whole group (percentage of all words chosen by whole group)	Number of words chosen by women (percentage of all words chosen by women)	Number of words chosen by men (percentage of all words chosen by men)
Food and drink	3071 (30.7%)	1162 (31.4%)	1909 (30.3%)
Nature and gardening	1024 (10.2%)	285 (7.7%)	739 (11.7%)
Entertainment	937 (9.4%)	296 (8%)	641 (10.2%)
Places	730 (7.3%)	235 (6.4%)	495 (7.9%)
People	671 (6.7%)	246 (6.7%)	425 (6.7%)
House	642 (6.4%)	260 (7.0%)	382 (6.1%)
Clothes	518 (5.2%)	278 (7.5%)	240 (3.8%)
Travel	345 (3.5%)	65 (1.7%)	283 (4.5%)
Actions	231 (2.3%)	71 (1.9%)	160 (2.5%)
Money and numbers	200 (2.0%)	110 (3.0%)	90 (1.5%)
Personal Care	200 (2.0%)	111 (3.0%)	89 (1.5%)
Shopping	196 (2.0%)	91 (2.5%)	105 (1.7%)
Time	190 (1.9%)	72 (1.9%)	118 (1.9%)
Health	167 (1.7%)	72 (1.9%)	95 (1.5%)
Work and education	162 (1.6%)	45 (1.2%)	117 (1.9%)
Personal items	151 (1.5%)	101 (2.7%)	50 (0.8%)
Other topics	564 (5.6%)	202 (5.5%)	362 (5.7%)
Total	9999 (100%)	3699 (100%)	6300 (100%)

Shading represents a difference off 50% or more between the percentages of words chosen from a topic in each subgroup.

**Table 4:** The number of words chosen from each topic by age.

Topic	Number of words chosen by whole group (percentage of all words chosen by whole group)	Number of words chosen by under65s (percentage of all words chosen by under65s)	Number of words chosen by participants 65 and over(percentage of all words chosen by participants 65 and over)
Food and drink	3071 (30.7%)	1162 (31.4%)	1909 (30.3%)
Nature and gardening	1024 (10.2%)	499 (9.6%)	525 (10.9%)
Entertainment	937 (9.4%)	501 (9.6%)	436 (9.1%)
Places	730 (7.3%)	397 (7.6%)	333 (6.9%)
People	671 (6.7%)	300 (5.8%)	371 (7.7%)
House	642 (6.4%)	347 (6.7%)	295 (6.1%)
Clothes	518 (5.2%)	271 (5.2%)	247 (5.1%)
Travel	345 (3.5%)	209 (4.0%)	136 (2.8%)
Actions	231 (2.3%)	143 (2.8%)	88 (1.8%)
Money and numbers	200 (2.0%)	131 (2.5%)	69 (1.4%)
Personal Care	200 (2.0%)	106 (2.0%)	94 (2.0%)
Shopping	196 (2.0%)	87 (1.7%)	109 (2.3%)
Time	190 (1.9%)	137 (2.6%)	53 (1.1%)
Health	167 (1.7%)	81 (1.6%)	86 (1.8%)
Work and education	162 (1.6%)	68 (1.3%)	94 (2.0%)
Personal items	151 (1.5%)	80 (1.5%)	79 (1.5%)
Other topics	564 (5.6%)	290 (5.6%)	274 (5.7%)
Total	9999 (100%)	5199 (100%)	4800 (100%)

Shading represents a difference of 50% or more between the percentages of words chosen from a topic in each subgroup.

Ninety four percent of words were selected from the top 16 topics, with each of these 16 constituting more than one percent of the total number of words. Table 3 shows that of those 16, nine of the topics were chosen with similar frequency by men and women with ‘food and drink’ representing 31 percent of the words chosen by women and 30 percent chosen by men. A greater percentage of words were selected from the topics ‘nature and gardening’ and ‘travel’ by men compared with women. A greater percentage of words from the topics ‘clothes’, ‘money and numbers’, ‘personal care’, ‘shopping’ and ‘personal items’ were chosen by woman compared with men. Table 4 shows that similar percentages of words were selected from each topic by participants under the age of 65 years and those who were aged 65 years or over for 13 of the 16 topics. ‘Travel’, ‘actions’ and ‘time’ were selected more often by those under the age of 65 than those aged 65 years and over.

Table 5 shows the ranking of topics per subgroup from highest to lowest with the highest ranking topic (1) being the topic with the largest percentage of word tokens selected from it and the lowest ranking (16) having the lowest percentage of word tokens selected from it. There was a higher percentage of words selected from the topic ‘food and drink’ than any other topic for all subgroups. ‘Nature and gardening’ and ‘entertainment’ ranked second or third across subgroups. Despite men choosing ‘nature and gardening’ words one and a half times as often as women, this topic still ranked in the top three for women. Although there is some difference in ranking position, Table 4 shows that the top eight ranked topics are ‘food and drink’, ‘nature and gardening’, ‘entertainment’, ‘places’, ‘people’, ‘house’, ‘clothes’ and ‘travel’ for all subgroups. The only exception is ‘travel’ which is not ranked in the top eight for women. The top eight categories represent 79.4 percent of word tokens chosen by the participants. There appears to be greater variation in the ranking of the less frequently chosen topics between subgroups.

**Table 5:** Ranking of topics by subgroup.

Ranking	Whole group	Women	Men	under65 years	65 years and above
1	Food and Drink	Food and Drink	Food and Drink	Food and Drink	Food and Drink
2	Nature and Gardening	Entertainment	Nature and Gardening	Entertainment	Nature and Gardening
3	Entertainment	Nature and Gardening	Entertainment	Nature and Gardening	Entertainment
4	Places	Clothes	Places	Places	People
5	People	House	People	House	Places
6	House	People	House	People	House
7	Clothes	Places	Travel	Clothes	Clothes
8	Travel	Personal care	Clothes	Travel	Travel
9	Actions	Money and numbers	Actions	Actions	Shopping
10	Money and numbers	Personal items	Time	Time	Personal care
11	Personal care	Shopping	Work and education	Money and numbers	Work and education
12	Shopping	Time	Shopping	Personal care	Actions
13	Time	Health	Health	Shopping	Health
14	Health	Actions	Money and numbers	Health	Personal items
15	Work and education	Travel	Personal care	Personal items	Money and numbers
16	Personal items	Work and education	Personal items	Work and education	Time

Table 2 shows the number of word types in each topic and Tables 3 and 4 show the number of word tokens selected from each topic. The topics with the highest number of word types (Table 2) and word tokens (Table 5) are similar but there are some differences noted in ranking. This is due to the fact that some topics have many word types each selected only a few times (such as people) whilst others have fewer word types each selected many times (such as clothes). For example the word ‘trousers’ was chosen several times by different participants.

### Distribution of words chosen from each topic across participants

**Fig 1:**
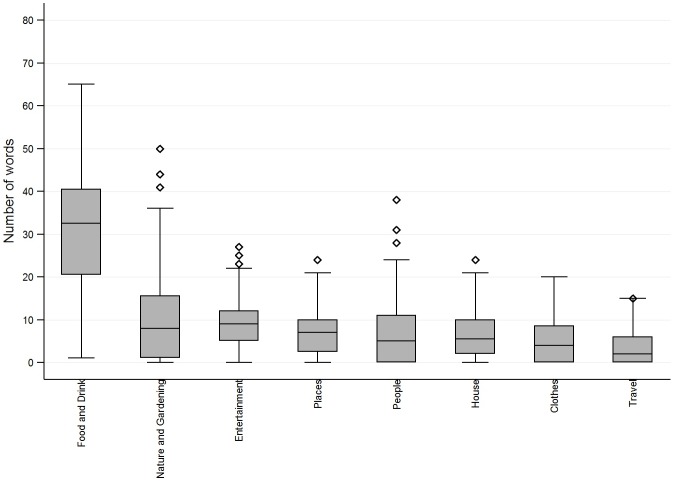
Distribution of words chosen across participants by topic.

Fig 1 shows the distribution of words chosen across participants for the eight highest ranking topics. ‘Food and drink’, from which the highest number of words was chosen, shows a normal distribution across participants. The number of words chosen from this topic was wide ranging from one to 65. For all other topics the median number of words chosen was less than 10 and the boxes are relatively short showing 50 percent of participants chose similar numbers of words from these topics. Not everyone chose words from these topics, however the longer upper whiskers show a spread of up to 37 words chosen from the topic ‘nature and gardening’, 20 to 25 words chosen from ‘entertainment’, ‘places’, ‘people’, ‘house’ and ‘clothes’, and up to 15 words chosen from ‘travel’. ‘Nature and gardening’, ‘entertainment’, ‘places’, ‘people’ and ‘house’ also show outliers (those more than one and a half times the interquartile range above the top of the interquartile range) representing participants who showed particular interest in these topics.

### Words chosen with the greatest frequency

**Fig 2:**
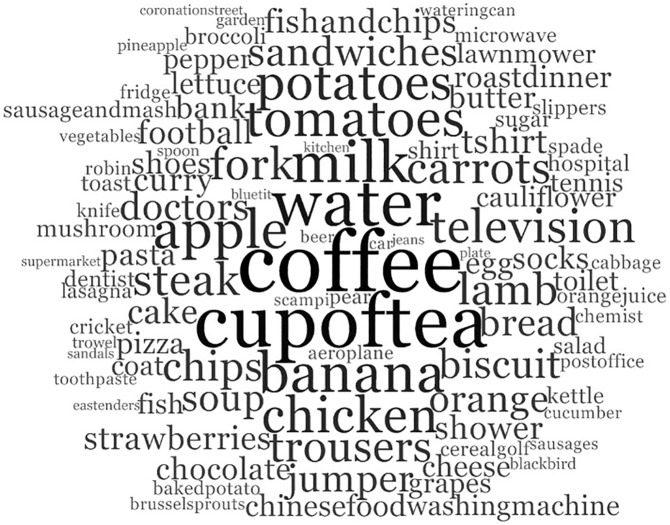
Words chosen with the greatest frequency.

Frequency of selection of each word type is shown in S3 File. Fig 2 illustrates the 102 most frequently selected words (more than one word was selected with the same frequency in one hundredth place). The frequency with which these words were chosen ranged from 18 percent (one in five participants) to 53 percent (one in two participants). This suggests that these words are likely to be chosen by other people with aphasia. Therefore pre-prepared pictures of these words could be used to save therapists time in creating them anew for each new patient. Indeed, these top 102 word types account for 27 percent of the 9999 words chosen by the 100 participants. ‘Coffee’ was chosen with the greatest frequency (58 percent of participants) closely followed by ‘cup of tea’ (53 percent of participants) and ‘water’ (48 percent of participants). The most frequently chosen words come from nine topics, with the majority (61 percent) related to food and drink. All of the topics correspond with the highest ranking topics except ‘people’. Words from the topic ‘people’ do not appear in the 102 most frequently selected words. This is likely to be because the greatest numbers of ‘people’ word types are people’s names which are unique to each individual and need a bespoke picture prepared in each case. For example, the name ‘John’ will represent a different person each time it is selected, requiring a different picture.

The words selected with the greatest frequency for each subgroup were identified and compared to the 102 words chosen with the greatest frequency by the whole group. Sixty percent of the 102 words were in the 100 (approximately) most frequently selected words for all subgroups. Of the most frequently chosen 51 words (two words were in fiftieth place) from the whole group, 90 percent appear in the 100 (approximately) most frequently selected words for all subgroups. Only five words in the 51 most frequently chosen words for the whole group were not frequently chosen by all subgroups: ‘football’, ‘tennis’ and ‘lawnmower’ were not in the top 100 words chosen by women; ‘washing machine’ was not in the top 100 words chosen by men; and ‘Chinese food’ was not in the top 100 words chosen by participants 65 years and over.

## Discussion

Inspired by a need to tailor stimuli for word finding therapy to be relevant to people with aphasia in a bid to maximise the impact of the therapy on their everyday lives, this paper reports on the content analysis of 9999 words selected as relevant by 100 people with aphasia. The sample of participants represents people with all severities of aphasia across a wide age range. The ratio of women to men (37:63) reflects the world-wide gender difference in stroke prevalence statistics [18]. Whereas 26 percent of strokes occur in people under the age of 65 years [17], a higher proportion (54 percent) of the sample in this study was under 65 years of age. This may be accounted for by higher survival rates amongst the younger age group. Additionally younger people may be more interested in participating in a research project involving treatment using a computer from which this cohort of participants was drawn. A subgroup analysis was conducted by age and gender to explore how generalisable the common topics and words from the whole group of participants are to these subgroups.

The words chosen by people with aphasia fell into 27 different topics. A subgroup analysis showed differences in the frequency with which words were selected from some of the topics. Men chose a greater percentage of words about ‘nature and gardening’, and ‘travel’ than women. By contrast women chose a greater percentage of words about ‘clothes’, ‘money and numbers’, ‘personal care’, ‘shopping’, and ‘personal items’ than men. Although the sample of participants in this study contained a greater proportion of under 65 year olds than expected in the stroke population, the percentage of words chosen by under 65 year olds and those 65 years and above was consistent for the majority of topics. Only three topics showed a difference between the age groups. Under 65 year olds chose a greater percentage of words about ‘time’, ‘actions’ and ‘travel’ than those 65 years old and above. Despite these differences between subgroups, the same eight topics are all ranked in the top eight for all subgroups (except for women, where ‘money and numbers’ was in eighth place and ‘travel’ fifteenth). This demonstrates relative consistency between subgroups. For example, although men chose a greater percentage of ‘nature and gardening’ words than women, the topic still ranks highly with both genders, being second for men and third for women. The top eight topics included ‘food and drink’, ‘nature and gardening’, ‘entertainment’, ‘places’, ‘people’, ‘house’, ‘clothes’ and ‘travel’ from which 79.4 percent of the words were selected. Different participants selected different numbers of words from these topics and some topics were of particular interest to a few individuals. However, there was a relatively normal distribution of words chosen from all of the top eight topics across participants.

Goals identified by people with aphasia (Worrall et al 2011) are reflected in these topics [19]. When looking at the frequency of words selected, ‘food and drink’ items were overwhelmingly popular, representing 30 percent or more of words chosen by all subgroups and 61 percent of the most frequently selected 102 words. One of the categories of goals Worrall et al (2011) identified was returning to pre-stroke life and that people with aphasia wanted ‘just to be normal and enjoy life’ [19] (p313). With food being a big part of everyday life and often the source of pleasure and social interaction, the desire to ‘just be normal and enjoy life’ may offer an explanation for the focus on talking about food and drink. Worrall et al (2011) stressed the importance of relationships to people with aphasia and noted that they often wanted to say specific names [19]. Two hundred and eighty three (9.1 percent) of the 3095 different word types selected in this study were names of specific people (excluding celebrities). Worrall et al (2011) also identified social goals including leisure and work as being particularly important once people were home from hospital: ‘it’s communication with other people…in the sense that…just to be talking to a neighbour’ [19] (p315). In addition, they identified the importance of talking about things that are connected to real life. Topics including ‘places’, ‘nature and gardening’, ‘house’, ‘shopping’, ‘clothes’, ‘personal items’, ‘personal care’, ‘health’, ‘weather’, ‘feelings and senses’, and ‘travel’ could all be considered to be ‘real life’ topics that could form the basis for conversation with family, friends and neighbours. The topic of ‘travel’ also contained words related to ‘holidays and trips’ which focus on leisure. One of the largest topics was ‘entertainment’ focussing entirely on leisure pursuits such as sports, hobbies, TV, films and music. Finally, Worrall et al (2011) showed that people with aphasia seek respect by highlighting their premorbid skills and accomplishments [19]. This desire may be represented by the topic ‘work and education’ and the technical and specialist vocabulary selected as words important for people with aphasia to say within several of the topics.

What any given person with aphasia identifies as being relevant personal vocabulary that would help them to meet their communication goals is of course unique to that individual. Therefore the speech and language therapist needs to prepare materials to stimulate the retrieval of a unique set of words for each person. This can be very time consuming if material for each word selected needs to be prepared anew. With the exception of pictures of specific people, which require bespoke preparation for each individual, words which can be represented by a common picture could be prepared in advance and used when requested by a new client. S3 File shows the words in order of the frequency with which they were selected for practice. Therapists could refer to this to decide which words to pre-prepare pictures for. This paper exemplified the 102 words selected with the highest frequency (chosen by between 18 and 58 percent of the whole participant group). It was demonstrated that the majority of these words were chosen with high frequency by all subgroups. These 102 words out of the total 3095 different word types accounted for 27 percent of the 9999 words chosen for practice. If therapists had pre-prepared resources which included these words it is likely that they would have easy access to materials for at least some of the words within an individual patient’s unique therapy set. This could reduce the time associated with preparation of personally relevant therapy material.

Deciding which words to focus on, in therapy for an individual, can be challenging for a number of reasons. People with more severe aphasia may find it difficult to identify or communicate the types of words that would make the most difference for them to be able to say. When a person with aphasia is still in hospital, they won’t yet have experienced the situations or topics of conversation that are difficult and most important for them to address in therapy. Therefore, being aware of words that other people with aphasia have identified as relevant may be a useful starting point to maximise the usefulness of early therapy or to create prompts to help people identify what is most relevant to them.

Limitations of this study and further research: The population studied were people with aphasia at least four months post stroke in the UK with a range of ages, aphasia severities and gender. Additional demographic details which may be relevant such as socio-economic group and ethnic origin are unknown. The subgroup analysis in this paper concentrated on demographics of age and gender. A future analysis could be conducted to explore differences between subgroups based on clinical severity of aphasia. One of the sources to prompt selection of words in this study was the existing large StepbyStep library of words. The large number of food and drink words selected led the authors to question whether this was due to a disproportionate number of food and drink words in the StepbyStep library. However, there were no more food and drink items represented in the library than words in other topic areas. The use of quantitative content analysis identifies categories of words that are important to people with aphasia but does not help us to understand why. The topics were related to goals identified by people with aphasia by Worrall et al (2011) to set them in a context [18]. However further qualitative research needs to be conducted to explore the reasons behind personal relevance of words and the links between the topics and rehabilitation goals.

## Conclusion

In conclusion, the study showed that words selected as personally relevant to people with aphasia fell into 27 different topic areas with participants most frequently selecting words from the topic ‘food and drink’. The study identified the frequency with which each word type was selected for practice by participants. The most frequently selected 102 words types accounted for 27 percent of the 9999 words tokens chosen for practice by the 100 participants. If pre-prepared resources contained the frequently selected words identified in this study, it is likely that they would contain some words selected by an individual with aphasia for therapy practice. This would reduce the time required in preparing personally relevant therapy material, facilitating therapy with relevant words to maximise the impact on everyday life.

## Supporting information

S1 File List of words selected by participants with aphasia. (CSV)

S2 File Participant demographics. (CSV)

S3 File Frequency with which words were selected. (CSV)
